# Supplementary material for: NanoBiT System and Hydrofurimazine for Optimized Detection of Viral Infection in Mice—A Novel in Vivo Imaging Platform
Source: Int J Mol Sci. 2020 Aug 15;21(16):5863. doi: 10.3390/ijms21165863 (PMC7461499; doi:10.3390/ijms21165863)
Supplement: Supplementary file 1 [file ijms-21-05863-s001.pdf]

International Journal of *Molecular Sciences*

MDPI

Article

NanoBiT System and Hydrofurimazine for Optimized Detection of Viral Infection in Mice−A Novel in Vivo Imaging Platform

Natasa Gaspar [1,2,3], Giorgia Zambito [1,2,4], Iris J.C. Dautzenberg [5], Steve J. Cramer [5], Rob C. Hoeben [5], Clemens Lowik [1,2,6], Joel R. Walker [7], Thomas A. Kirkland [7], Thomas P. Smith [7], Wytske M. van Weerden [8], Jeroen de Vrij [9] and Laura Mezzanotte [1,2,*]

1. Department of Radiology and Nuclear Medicine, Erasmus Medical Center, 3015 GD Rotterdam, Netherlands; n.gaspar@erasmusmc.nl (N.G.); g.zambito@erasmusmc.nl (G.Z.); c.lowik@erasmusmc.nl (C.L.); l.mezzanotte@erasmusmc.nl (L.M.)
2. Erasmus Medical Center, Department of Molecular Genetics, 3015 GD Rotterdam, Netherlands
3. Percuros B.V., 2333 CL Leiden, The Netherlands
4. Medres Medical Research GMBH, 50931 Cologne, Germany
5. Leiden University Medical Center, Department of Cell and Chemical Biology, 2333 Leiden, Netherlands; I.J.C.Dautzenberg@lumc.nl (I.J.C.D.); s.j.cramer@lumc.nl (S.J.C.); r.c.hoeben@lumc.nl (R.C.H.)
6. Department of Oncology CHUV, UNIL and Ludwig Cancer Center, 1011 Lausanne, Switzerland
7. Promega Biosciences L.L.C., San Luis Obispo, 93401 California, United States of America; Joel.Walker@promega.com (J.R.W.); thomas.kirkland@promega.com (T.A.K.); thomas.smith@promega.com (T.P.S.)
8. Erasmus Medical Center, Department of Urology, 3015 GD Rotterdam, Netherlands; w.vanweerden@erasmusmc.nl
9. Erasmus Medical Center, Department of Neurosurgery, 3015 GD Rotterdam, Netherlands; j.devrij@erasmusmc.nl

* Correspondence: l.mezzanotte@erasmusmc.nl; Tel.: +31634681193

Received: 16 July 2020; Accepted: 03 August 2020; Published: date

Supplementary Figure 1

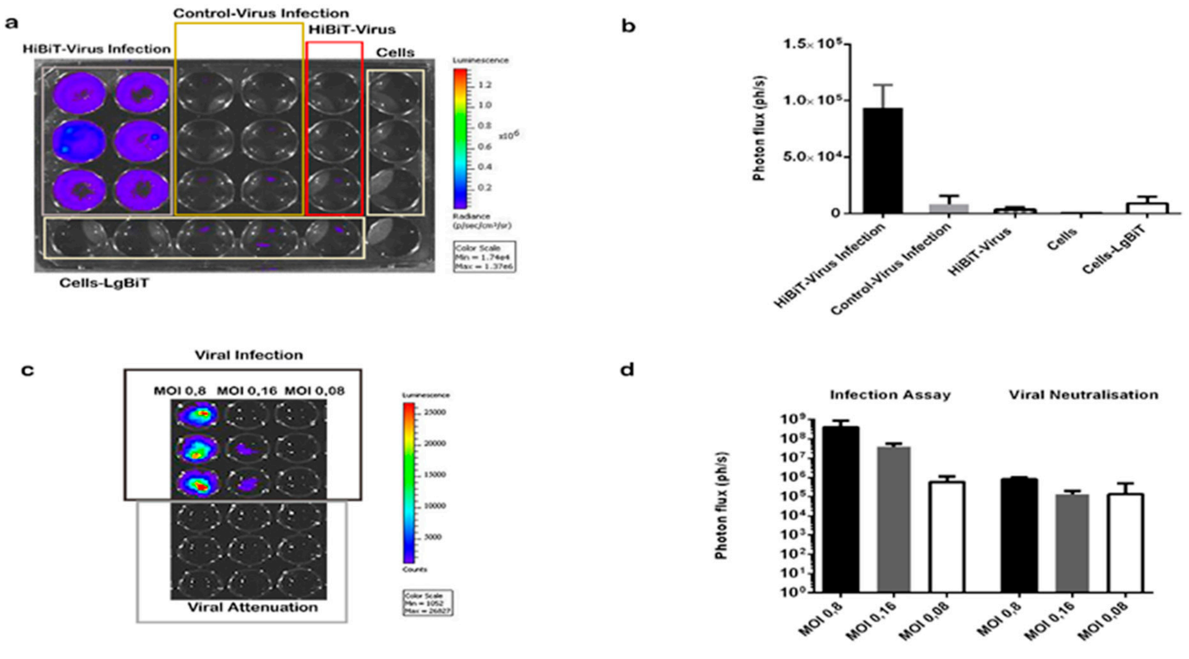

Supplementary Figure 1. NanoBiT system applied with different cell lines. (a) PC339C, expressing LgBiT were infected with the HiBiT-reporter virus or the control virus not containing the HiBiT tag. The infected cells were imaged 24 h post infection by addition of substrate from the Nano-Glo Luciferase Assay System with a final concentration of 0.1 mM. We show as well the minimum background signal coming from the infection of cells (PC339C-LgBiT) with the control virus; the PC339C cells on their own; the PC339C-LgBiT cells; HiBiT-reporter virus post substrate addition. (b) Signals were quantified with IVIS software. Quantification of detected signal as correlation between detected light and viral infectivity after background subtraction. Results are presented as means +SD. **(c)** PC346C cells expressing LgBiT were plated in 96 well plates and were infected with HiBiT-reporter virus dilutions of initial viral stock (ranging from 0.8 to 0.08 MOI) or with the neutralized HiBiT-reporter virus by pre-incubation with intravenous immunoglobulin G (IvIg) with a final concentration of 50 mg mL^{-1}. The infected cells were imaged 24 h post infection by addition of substrate from the Nano-Glo Luciferase Assay System with a final concentration of 0.1 mM. **(d)** Signals were quantified with IVIS software. Quantification of detected signal as correlation between detected light and viral infectivity after background subtraction. Results are presented as means +SD.
